# Novel Highly Pathogenic Avian A(H5N2) and A(H5N8) Influenza Viruses of Clade 2.3.4.4 from North America Have Limited Capacity for Replication and Transmission in Mammals

**DOI:** 10.1128/mSphere.00003-16

**Published:** 2016-04-06

**Authors:** Bryan S. Kaplan, Marion Russier, Trushar Jeevan, Bindumadhav Marathe, Elena A. Govorkova, Charles J. Russell, Mia Kim-Torchetti, Young Ki Choi, Ian Brown, Takehiko Saito, David E. Stallknecht, Scott Krauss, Richard J. Webby

**Affiliations:** a Department of Infectious Diseases, St. Jude Children’s Research Hospital, Memphis, Tennessee, USA; b Department of Population Health, College of Veterinary Medicine, The University of Georgia, Athens, Georgia, USA; c National Veterinary Services Laboratories, Science, Technology and Analysis Services, Veterinary Services, Animal and Plant Health Inspection Service, U.S. Department of Agriculture, Ames, Iowa, USA; d College of Medicine and Medical Research Institute, Chungbuk National University, Cheongju, South Korea; e Animal and Plant Health Agency, New Haw, Addlestone, Surrey, United Kingdom; f Influenza and Prion Disease Research Center, National Institute of Animal Health, National Agriculture and Food Research Organization, Tsukuba, Ibaraki, Japan; Emory University School of Medicine

**Keywords:** avian influenza virus, H5N2, H5N8, mammals, viral replication

## Abstract

Highly pathogenic influenza A(H5N8) viruses from clade 2.3.4.4 were introduced to North America by migratory birds in the fall of 2014. Reassortment of A(H5N8) viruses with avian viruses of North American lineage resulted in the generation of novel A(H5N2) viruses with novel genotypes. Through sequencing of recent avian influenza viruses, we identified PB1 and NP gene segments very similar to those in the viruses isolated from North American waterfowl prior to the introduction of A(H5N8) to North America, highlighting these bird species in the origin of reassortant A(H5N2) viruses. While they were highly virulent and transmissible in poultry, we found A(H5N2) viruses to be low pathogenic in mice and ferrets, and replication was limited in both hosts compared with those of recent highly pathogenic avian influenza (HPAI) H5N1 viruses. Molecular characterization of the hemagglutinin protein from A(H5N2) viruses showed that the receptor binding preference, cleavage, and pH of activation were highly adapted for replication in avian species and similar to those of other 2.3.4.4 viruses. In addition, North American and Eurasian clade 2.3.4.4 H5NX viruses replicated to significantly lower titers in differentiated normal human bronchial epithelial cells than did seasonal human A(H1N1) and highly pathogenic A(H5N1) viruses isolated from a human case. Thus, despite their having a high impact on poultry, our findings suggest that the recently emerging North American A(H5N2) viruses are not expected to pose a substantial threat to humans and other mammals without further reassortment and/or adaptation and that reassortment with North American viruses has not had a major impact on viral phenotype.

**IMPORTANCE** Highly pathogenic H5 influenza viruses have been introduced into North America from Asia, causing extensive morbidity and mortality in domestic poultry. The introduced viruses have reassorted with North American avian influenza viruses, generating viral genotypes not seen on other continents. The experiments and analyses presented here were designed to assess the impact of this genetic diversification on viral phenotypes, particularly as regards mammalian hosts, by comparing the North American viruses with their Eurasian precursor viruses.

## Abstract (precis)

Highly pathogenic H5 influenza viruses have been introduced into North America from Asia, causing extensive morbidity and mortality in domestic poultry. The introduced viruses have reassorted with North American avian influenza viruses, generating viral genotypes not seen on other continents. The experiments and analyses presented here were designed to assess the impact of this genetic diversification on viral phenotypes, particularly as regards mammalian hosts, by comparing the North American viruses with their Eurasian precursor viruses.

## INTRODUCTION

Highly pathogenic avian influenza (HPAI) viruses of the A/goose/Guangdong/1/1996 H5N1 lineage have loomed as a pandemic threat since the first human case was reported in 1997 (1, 2). In the two decades following the first human cases, these viruses have undergone geographic spread and genetic divergence of the A/goose/Guangdong/1/1996 lineage hemagglutinin (HA) into many phylogenetic clades. They have since become enzootic in the domestic poultry populations of many countries across Asia and the Middle East and continue to cause sporadic infections in humans.

In January 2014, outbreaks of a novel HPAI caused by A(H5N8) viruses of HA clade 2.3.4.4 were reported in poultry and wild birds in eastern China and South Korea (3–5). In November 2014, closely related viruses were detected in wild and domestic bird species in North America, signaling the first detection of the A/goose/Guangdong/1/1996 lineage viruses on the continent (6). Subsequently, reassortment with avian influenza viruses circulating in North America led to the emergence of HPAI A(H5N2) viruses that became widespread across the United States and Canada and have been responsible for the culling of millions of domestic poultry (7–9).

As with all emerging HPAI viruses, understanding the potential risks to humans is a priority. Reassortment of influenza A viruses (IAV) from different lineages can result in viruses with unpredictable phenotypes as highlighted by the reassortment of Eurasian and North American swine viruses that gave rise to the 2009 pandemic A(H1N1) virus (10, 11). In addition, human cases of A/goose/Guangdong/1/1996 lineage A(H5N6) viruses of clade 2.3.4.4 have been reported in China (12, 13), demonstrating that these viruses can infect humans. In this study, we aimed to understand the impact that reassortment of clade 2.3.4.4 A(H5N8) viruses with North American avian viruses has had on the pathogenicity and transmissibility of newly emerged A(H5N2) HPAI viruses in mammals.

## RESULTS

### Source of clade 2.3.4.4 A(H5N2) North American lineage influenza virus genes.

**FIG 1:**
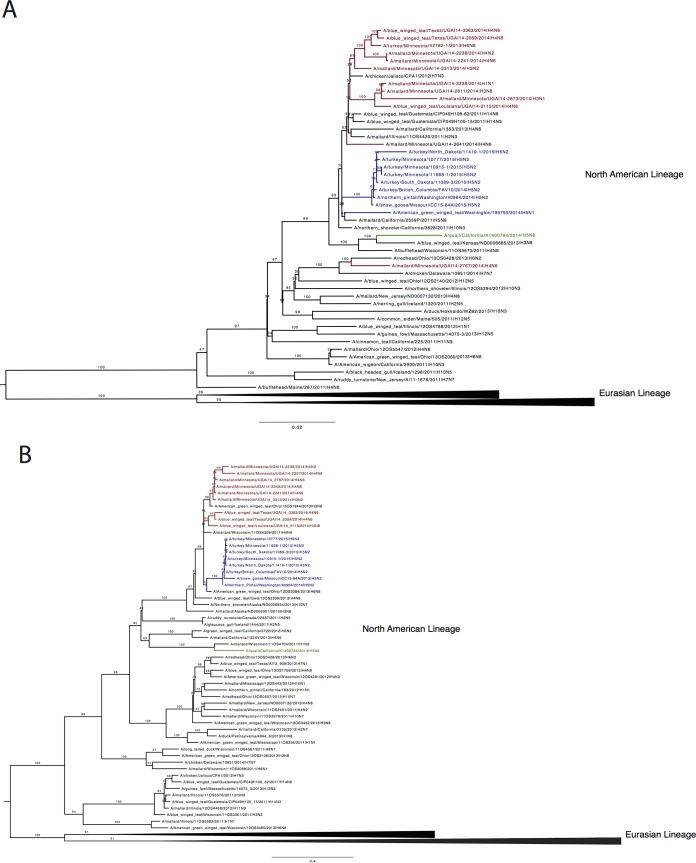
Phylogenetic relationships of the PB1 and NP gene segments of North American clade 2.3.4.4 A(H5N2) avian influenza viruses. Maximum-likelihood phylogenetic trees illustrating the relationships of PB1 (A) and NP (B) gene segments from clade 2.3.4.4 A(H5N2) strains. Bootstrap values (*n* = 1,000) for all nodes are provided. Trees are rooted on Eurasian lineage branches (collapsed). A(H5N2) gene segments are labeled in blue, recent low-pathogenic wild bird gene segments are in red, and LPAI Ql/CA/14, used in experimental studies, is labeled in green.

The North American clade 2.3.4.4 A(H5N2) viruses are reassortants incorporating PB1, NP, and NA gene segments from North American avian viruses, with the remaining gene segments contributed by Eurasian A(H5N8) viruses (8). Here, we sought to provide greater granularity on the likely avian source of the North American gene segments. To achieve this, we sequenced a number of recent avian viruses randomly selected from our ongoing surveillance activities in wild birds in the Mississippi flyway (Table 1). We identified closely related PB1 (>97% sequence homology [see Table S1 in the supplemental material]) and NP (>98% sequence homology [see Table S2]) gene segments in viruses isolated from mallard ducks (*Anas platyrhynchos*) and green winged teal (*Anas carolinensis*) (Fig. 1A and B), though we failed to identify closely related NA gene segments (data not shown). The closely related PB1 gene segments were detected in H1 to H4 IAV collected from mallards, in Minnesota, and teal, in Louisiana and Texas, during 2014. The NP gene segments also clustered closely with NP segments from H3 to H6 viruses sampled from mallards (Minnesota) and teal (Louisiana and Texas). Ancestral PB1 and NP gene segments were identified in North American specimens from at least 2011 onward in both the Pacific and Mississippi flyways. These results implicate North American waterfowl (mallards and teals) as the source of A(H5N2) reassortant gene segments. Additionally, these data show the utility of influenza surveillance networks in the detection and characterization of emerging, potentially pandemic viruses.

**TABLE 1:** List of viruses, and their abbreviations, used in this study

Subtype	Virus	Abbreviation	Presence of MBS^a^
H5N8	A/mallard/Korea/W452/2014	Md/Korea/14	Yes
	A/chicken/Kumamoto/1/7/2014	Ck/Kumamoto/14	Yes
	A/duck/England/36254/2014	Dk/England/14	Yes
	A/gyrfalcon/Washington/41088-6/2014	Gyr/WA/14	Yes
H5N2	A/Northern pintail/Washington/40964/2014	NP/WA/14	Yes
	A/snow goose/Missouri/CC15−84A/2015	SG/MO/15	Yes
	A/turkey/Minnesota/11688-1/2015	Tk/MN688/15	Yes
	A/turkey/Minnesota/10777/2015	Tk/MN777/15	Yes
	A/turkey/Minnesota/10915-1/2015	Tk/MN915/15	Yes
	A/turkey/South Dakota/11089-3/2015	Tk/SD/15	Yes
	A/turkey/North Dakota/11419-1/2015	Tk/ND/14	Yes
H1N1	A/CA/04/2009 H1N1	CA/09	No
H1N1	A/Tennessee/1-590/2009	TN/09	No
H5N1	A/Vietnam/1203/2004 H5N1	VN/04	Yes
H5N8	A/quail/CA/K1400794/2014 H5N8	Ql/CA/14	No
H5N2	A/ruddy turnstone/Delaware/431/2011	RT/DE/11	No

a Hemagglutinin protein contains a multibasic cleavage site (MBS).

10.1128/mSphere.00003-16.1 Table S1  Nucleic acid sequence identity of clade 2.3.4.4 A(H5N2) virus PB1 gene segments. Download Table S1, DOCX file, 0.02 MB. Copyright © 2016 Kaplan et al. 2016 Kaplan et al. This content is distributed under the terms of the Creative Commons Attribution 4.0 International license.

10.1128/mSphere.00003-16.2 Table S2  Nucleic acid sequence identity of clade 2.3.4.4 A(H5N2) virus NP gene segments. Download Table S2, DOCX file, 0.1 MB. Copyright © 2016 Kaplan et al. 2016 Kaplan et al. This content is distributed under the terms of the Creative Commons Attribution 4.0 International license.

### Properties of the HA protein of clade 2.3.4.4 A(H5N2) and A(H5N8) influenza viruses.

The hemagglutinin (HA) protein of influenza A viruses is known to contribute to host range, pathogenicity, and transmissibility in avian and mammalian species.

**FIG 2:**
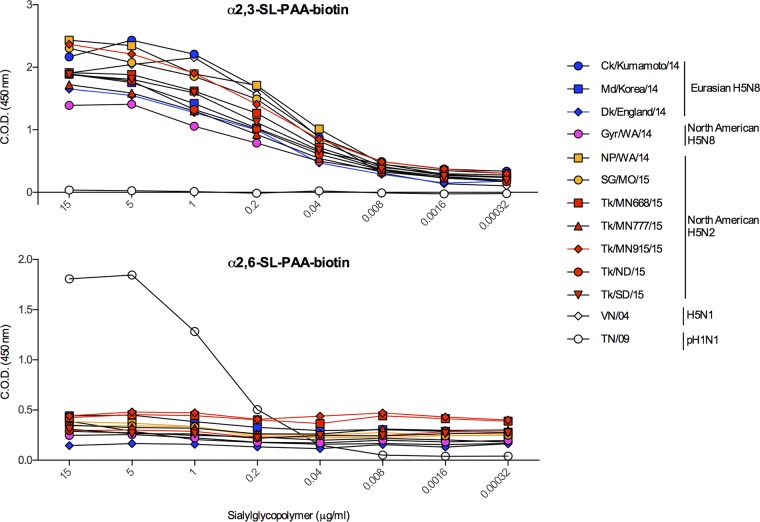
Receptor binding preference of HPAI A(H5N8) and A(H5N2) viruses. The receptor binding specificity of the HA protein of A(H5N8) and A(H5N2) viruses for α2,3-SA or α2,6-SA was analyzed by solid-phase assay. Pdm(H1N1) and HPAI A(H5N1) viruses were used as controls for α2,6- and α2,3-SA preference, respectively. Bound viruses were incubated with serial dilutions of biotinylated sialylglycopolymers: 3′-sialyllactose (3′SL) (α2,3-SL-phosphonoacetic acid [PAA]-biotin) and 6′-sialyllactosamine (6′SLN) (α2,6-SL-PAA-biotin). The absorbance was quantified and represents a measure of the strength of binding of the virus to the glycans. The results are shown as the means from 2 replicates. C.O.D., corrected optical density.

Contributing factors, namely, the binding preference for galactose-linked sialic acids (SA), requirement of proteases for cleavage activation, and pH sensitivity of acid-triggered membrane fusion, must display the correct phenotype for virus entry into the cell of a particular host species. To characterize the receptor binding preference of clade 2.3.4.4 H5NX viruses, we ran a solid-phase binding assay with plates coated with α2,3-SA (avian-like receptor) and α2,6-SA (human-like receptor). All A(H5N8) and A(H5N2) viruses of North American and Eurasian origin tested preferentially bound to α2,3-SA (avian-like receptor), with minimal binding to α2,6-SA (human-like receptor) (Fig. 2), indicating that the HA of these viruses binds weakly to the dominant receptor of the human upper respiratory tract, α2,6-SA, which is a barrier to human infection.

**FIG 3:**
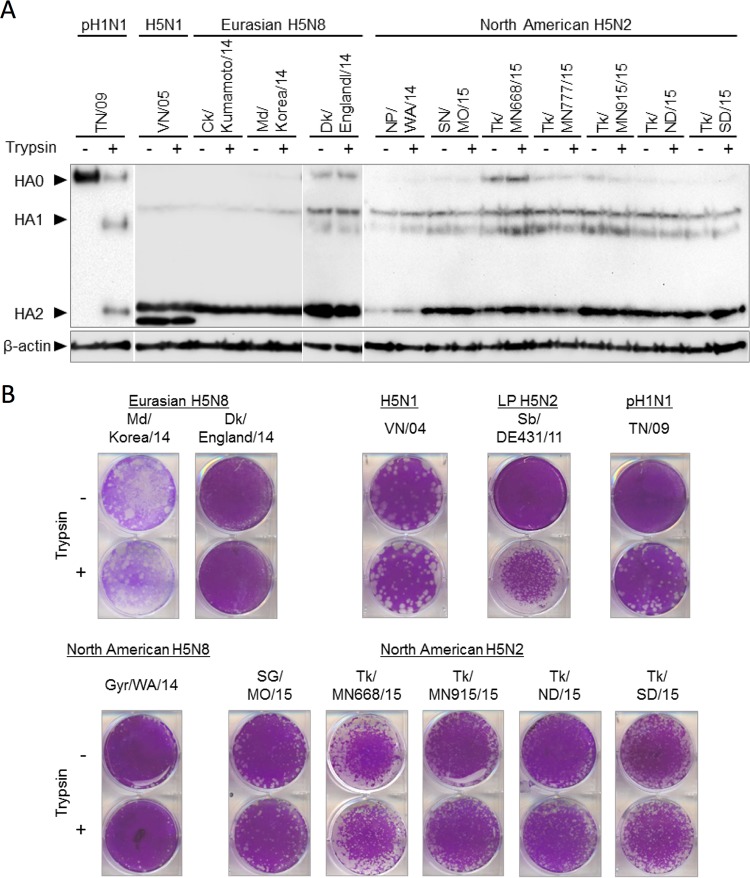
Cleavability of HA protein from clade 2.3.4.4 A(H5N8) and A(H5N2) viruses. (A) Vero cells were infected with A(H5N8) or A(H5N2) virus. A(H5N1) and Pdm(H1N1) infections were used as controls for the presence and absence of HA cleavage without exogenous trypsin, respectively. At 24 hpi, cells were treated (+) or not treated (−) with trypsin for 15 min. The lysates were resolved on an SDS-PAGE gel and further analyzed by Western blotting using ferret antisera. β-Actin was chosen as a housekeeping gene. (B) The ability of A(H5N8) and A(H5N2) viruses to form plaques in the absence of trypsin was evaluated by plaque assay in MDCK cells.

The cleavability of the HA protein is a major determinant for IAV pathogenicity. Highly pathogenic avian influenza (HPAI) viruses possess a unique multibasic cleavage motif in the connecting peptide region of the HA protein that allows cleavage of the HA0 precursor without extracellular protease (14). To better understand the pathogenicity of the emerging North American A(H5N8) and A(H5N2) viruses, we characterized the cleavage properties of the HA protein in the presence and absence of exogenous protease via Western blotting and plaque assay. As expected due to the presence of a multibasic cleavage site, the HA proteins of all Eurasian A(H5N8), North American A(H5N8), and North American A(H5N2) viruses were susceptible to cleavage *in vitro* in the absence of exogenous trypsin (Fig. 3A and B). This is similar to HPAI H5N1 but in contrast to human H1N1 virus.

**FIG 4:**
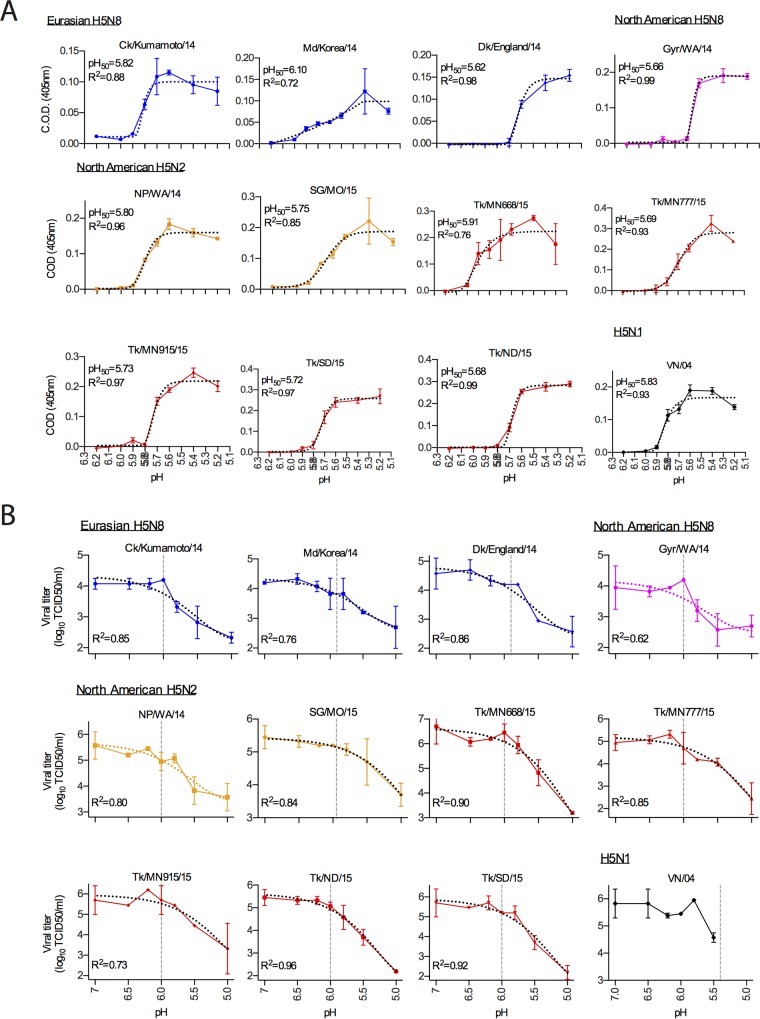
Acid stability of the HA protein of clade 2.3.4.4 A(H5N8) and A(H5N2) viruses. (A) Acid-induced hemolysis was determined using cRBC. The viruses were bound to cRBC, and hemolysis was induced by treatment with different pH-adjusted buffers. The corrected optical density (C.O.D.) measures the amount of hemoglobin released after fusion. The results are shown as means ± standard deviations from 4 replicates at the indicated pH value. The curves were fitted to an asymmetrical (five-parameter) regression model (dotted line), and the pH_50_ values were determined as the point at which a 50% change between maximum and baseline was observed. (B) Acid-induced inactivation of A(H5N8) and A(H5N2) viruses. The viruses were treated with different pH-adjusted buffers, and the remaining infectious virions were titrated by TCID_50_. The results are shown as means ± standard deviations from 2 replicates at the indicated pH value. The curves were fitted to an asymmetrical (five-parameter) regression model (dotted line), and the pH_50_ values were determined as the point at which a 50% change between maximum and baseline was observed.

During virus entry, following HA binding to its cognate receptor, influenza virions are endocytosed where the HA protein undergoes irreversible structural changes due to the acidification of the endosomal lumen. Endosomal acidification results in HA-mediated fusion of virion and endosomal membranes, releasing the viral genome into the host cell cytoplasm. As previously described, acid stability contributes to avian versus human infection and transmissibility, where increased acid stability (low activation pH, 5.0 to 5.5) is required for human infection (15–18). We measured the HA activation pH values of clade 2.3.4.4 viruses to further identify the risk of transmission to humans. We observed that all A(H5N8) and A(H5N2) viruses tested, regardless of continent of origin, had a high activation pH (5.9 to 6.0) as determined by syncytium assay (Table 2). This is consistent with the range of pH values for HPAI H5N1 viruses (5.6 to 6.0), while a low pH of HA activation (5.0 to 5.5) correlates with human adaptation (reviewed in reference 19). Additionally, in hemolysis assays the midpoint pH for membrane disruption (a readout of HA protein activation) was 5.6 to 6.1 for Eurasian H5N8 viruses, 5.7 for the North American H5N8 virus, and 5.7 to 5.9 for the North American H5N2 viruses (Fig. 4A). This is similar to A(H5N1) VN/04 (pH 5.8), which is readily transmissible in avian species but not in mammals (18, 20). Finally, we showed that the emerging A(H5N8) and A(H5N2) viruses were as equally sensitive to acid inactivation as was the HPAI VN/04 A(H5N1) virus. Acid inactivation studies found that 50% inactivation of virus occurred at pH values ranging from 5.6 to 5.7, 5.8, and 5.2 to 5.7 for Eurasian A(H5N8), North American A(H5N8), and North American A(H5N2) viruses, respectively (Fig. 4B). Together, these data show that the newly emerged A(H5N8) and A(H5N2) viruses have characteristics similar to those of other HA clades of HPAI viruses, including cleavage pattern, receptor binding preference, and fusion properties, suggesting that the HA proteins of these viruses are poorly adapted for infection and transmission in mammals.

**TABLE 2:** HA activation pH of clade 2.3.4.4 A(H5N2) and A(H5N8) viruses determined by syncytium assay

Virus	Subtype	pH^a^								pH of activation
		5.2	5.4	5.6	5.7	5.8	5.9	6.0	6.2	
Ck/Kumamoto/14	H5N8	+	+	+	+	+	+	+	−	6.0
Md/Korea/14	H5N8	+	+	+	+	+	+	−	−	5.9
Dk/England/14	H5N8	+	+	+	+	+	+	−	−	5.9
Gyr/WA/14	H5N8	+	+	+	+	+	+	+	−	6.0
NP/WA/14	H5N2	+	+	+	+	+	+	+	−	6.0
SG/MO/15	H5N2	+	+	+	+	+	+	+/−	−	5.95
Tk/ND/15	H5N2	+	+	+	+	+	+	+	−	6.0
Tk/SD/15	H5N2	+	+	+	+	+	+	+	−	6.0
Tk/MN668/15	H5N2	+	+	+	+	+	+	+	−	6.0
Tk/MN777/15	H5N2	+	+	+	+	+	+	+	−	6.0
Tk/MN915/15	H5N2	+	+	+	+	+	+	+	−	6.0
VN/04	H5N1	+	+	+	+	+	+	+	−	6.0
TN/09	H1N1	+	+	−	−	−	−	−	−	5.4

a + and − indicate the presence or absence of syncytium, respectively. Vero cells were infected with H5N8 and H5N2 viruses. Pandemic 2009 H1N1 virus was used as a control for optimal pH for human IAV.

### Susceptibility of A(H5N2) and A(H5N8) influenza viruses of clade 2.3.4.4 to NAIs.

**FIG 5:**
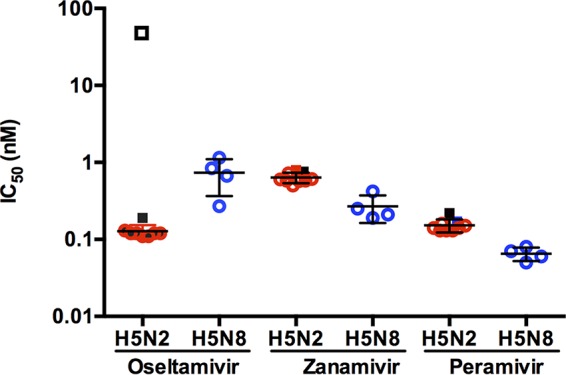
Inhibitory concentrations (IC_50_) of neuraminidase inhibitors (NAIs) against clade 2.3.4.4 IAV. Standardized quantities of neuraminidase activity were incubated with oseltamivir, peramivir, and zanamivir and assessed for their ability to metabolize MUNANA. A(H5N2) (red outline) and A(H5N8) (blue outline) strains are highly susceptible to NAIs. Oseltamivir-sensitive A/Fukuda/45/2004 (H3N2) (black filled square); oseltamivir-resistant A/Fukuda/20/2004 (H3N2) E119V (black outlined square); and oseltamivir (not shown)-, zanamivir-, and peramivir-resistant A/Shanghai/1/2013 (H7N9) R292K (green outlined square) were included for comparison.

The neuraminidase inhibitors (NAIs) are the only class of antiviral drugs currently recommended for the treatment of influenza virus infections. However, as with all virus protein-targeted drugs, NAI-resistant viruses can emerge either under drug treatment or naturally during virus evolution (21, 22). Susceptibility of novel IAV strains to antiviral compounds is one of 10 criteria composing the Influenza Risk Assessment Tool (IRAT), a scale that serves to quantify the risk of zoonotic influenza virus strains to humans (23). As such, we assessed the efficacy of antiviral interventions against A(H5N2) and A(H5N8) influenza A viruses by evaluating their susceptibility to three NAIs (oseltamivir, zanamivir, and peramivir) in the phenotypic NA enzyme inhibition assay with MUNANA [2′-(4-methylumbelliferyl)-α-d-*N*-acetylneuraminic acid] substrate (Fig. 5).

Influenza A(H5N2) and A(H5N8) viruses were fully susceptible to all three NAIs tested with mean 50% inhibitory concentrations (IC_50_s) no greater than 1.0 nM. The mean IC_50_s for oseltamivir, zanamivir, and peramivir determined for A(H5N2) and A(H5N8) viruses were comparable to those obtained with NAI-susceptible A/Fukuda/45/2004 (H3N2) virus (mean IC_50_, 0.19; 0.76 and 0.17 nM, respectively) and were significantly lower than the mean IC_50_ (50.83 nM) of oseltamivir-resistant A/Fukuda/20/2004 (H3N2) virus with E119V NA substitution. Overall, influenza A(H5N2) viruses were more susceptible to oseltamivir than were A(H5N8) viruses (mean IC_50_s, 0.12 and 0.61 nM, respectively). The IC_50_s for zanamivir and peramivir were 2-fold higher for A(H5N2) viruses than for A(H5N8) viruses. Taken together, these data suggest that NAI will be a viable treatment option for the infections caused by A(H5N2) and A(H5N8) viruses. Additionally, susceptibility to adamantanes was determined by amino acid analysis of M2 protein for known molecular markers of resistance. All A(H5N2) and A(H5N8) viruses encode asparagine at position 31 of the M2 protein (data not shown), a mutation known to confer resistance to adamantanes (22), indicating that these viruses are resistant to M2 ion channel blockers.

### Replication of A(H5N2) and A(H5N8) in differentiated NHBE cells.

**FIG 6:**
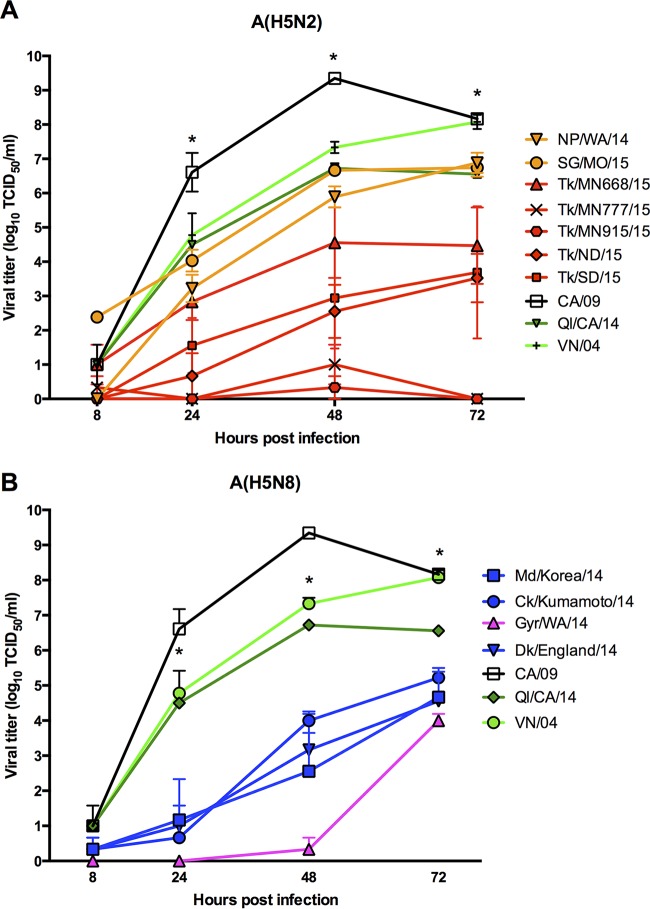
Replication kinetics of A(H5N8) and A(H5N2) viruses in differentiated NHBE cells. Replication kinetics of A(H5N2) (A) and A(H5N8) (B) virus strains in differentiated NHBE cells. NHBE cells were inoculated at an MOI of 0.01, and samples were collected at 8, 24, 48, and 72 hpi. Clade 2.3.4.4 A(H5N8) strains showed delayed replication compared to A(H5N2) strains NP/WA/14, SG/MO/15, LPAI Ql/CA/14, human pandemic CA/09, and human HPAI VN/04. All clade 2.3.4.4 viruses replicated to significantly (two-way analysis of variance; *, *P* < 0.05) lower titers than did CA/09 and VN/04.

To assess the ability of A(H5N2) and A(H5N8) influenza viruses to replicate in primary human cells, we performed multistep growth curves in differentiated normal human bronchial epithelial (NHBE) cells (Fig. 6). Virus titers determined after inoculation of NHBE cells with A(H5N2) and A(H5N8) viruses were significantly lower (*P* < 0.05) than those after inoculation with the 2009 pandemic H1N1 virus CA/09 (pH1N1) and HPAI strain VN/04 (H5N1) viruses (10^8^ to 10^9^ 50% tissue culture infective doses [TCID_50_]/ml). A(H5N2) viruses showed multiple replication phenotypes on differentiated NHBE cells; NP/WA/14 and SG/MO/15 reached appreciable titers (10^6^ TCID_50_/ml) that were significantly higher (*P* < 0.05) than those of TK/MN668/15 (10^4^ TCID_50_/ml) and the remaining clade 2.3.4.4 A(H5N2) viruses examined. In contrast, the replication of A(H5N8) viruses was delayed and titers reached were significantly lower (10^3^ to 10^4^ TCID_50_/ml) than those of the control viruses (CA/09 and VN/04). Interestingly, the North American low-pathogenic avian influenza (LPAI) Ql/CA/14 (H5N8) virus showed replication kinetics similar to those of VN/04 and reached appreciable titers (10^6.5^ TCID_50_/ml) similar to peak titers attained by NP/WA/14 and SG/MO/15 (10^6^ TCID_50_/ml). These results indicate that clade 2.3.4.4 H5NX viruses are compromised in their ability to replicate in primary human cells compared to human A(H1N1) and HPAI A(H5N1).

### Pathogenicity of A(H5N2) and A(H5N8) influenza viruses of clade 2.3.4.4 in mice.

**FIG 7:**
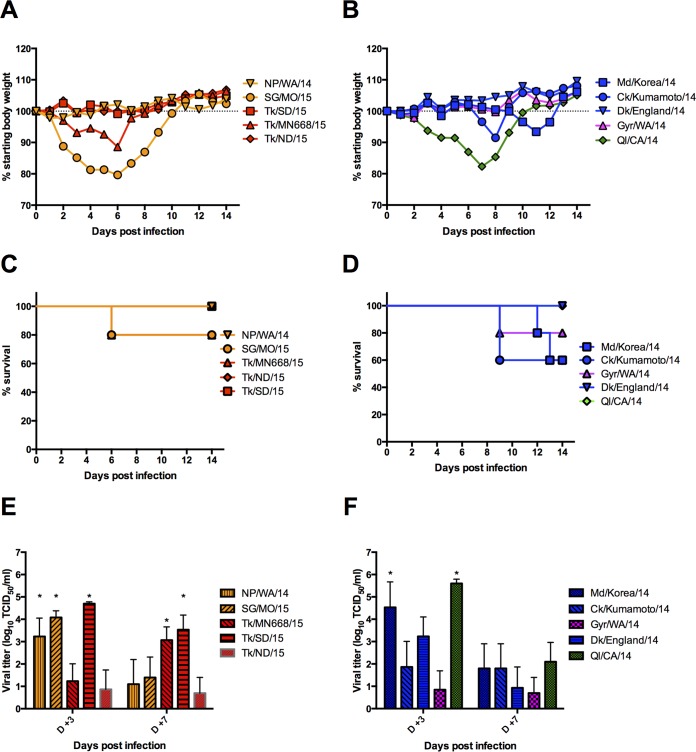
Pathogenicity of A(H5N8) and A(H5N2) viruses in mice. Fifteen C57BL/6 mice per virus were inoculated with 10^6^ EID_50_ of allantoic fluid in PBS intranasally. Five mice per virus were euthanized at 3 and 7 dpi for organ collection and virus titration, while the remaining five mice were observed for weight loss and mortality for 14 dpi. There was no significant difference in weight lost by C57BL/6 mice infected with 2.3.4.4 H5N2 (A) and H5N8 (B) compared to LPAI Ql/CA/14. Kaplan-Meier survival curves of mice infected with A(H5N8) (D) show Ck/Kumamoto/14 and Md/Korea/14 to be slightly more lethal (40% mortality) than other A(H5N8) viruses and A(H5N2) viruses (C) (≤20%). Lung virus titers from A(H5N2) (E)- and A(H5N8) (F)-inoculated mice show low levels of virus replication in all groups. Md/Korea/14, NP/WA/14, SG/MO/15, and Tk/SD/15 titers were significantly higher (*, *P* < 0.05) than those of other clade 2.3.4.4 strains, though not significantly different from the lung virus titers of Ql/CA/14-infected mice.

To assess the impact of reassortment on the pathogenicity of the clade 2.3.4.4 viruses in mice, C57BL/6 mice were inoculated intranasally (10^6^ 50% egg infective doses [EID_50_]/30 µl) with select viruses. Mice infected with Eurasian HPAI A(H5N8) viruses (Md/Korea/14 and Ck/Kumamoto/14) showed moderate weight loss (~10% average initial body weight) and an increased, though not significant, difference in mortality (40%) compared to Gyr/WA/14 (20% mortality) and LPAI A(H5N8) viruses (Fig. 7B and D). In contrast, weight loss was more pronounced in mice inoculated with the A(H5N2) strains, SG/MO/15 and TK/MN668/15 (10 to 20% average initial body weight), although no increase in mortality (20%) was observed following SG/MO/15 and Tk/SD/15 infection (Fig. 7A and C).

Both A(H5N2) and A(H5N8) virus isolates were capable of replication in the lungs of inoculated mice, although titers were modest (Fig. 7E and F). Of the HPAI A(H5N8) viruses used in murine inoculations, the Eurasian isolates Md/Korea/14, Ck/Kumamoto/14, and Dk/England/14 replicated to higher titers (2.33 to 5.66 log TCID_50_/ml) than did Gyr/WA/14 (1.06 log TCID_50_/ml) at 3 days postinoculation (dpi). Reduced lung virus titers were observed at 7 dpi in all HPAI A(H5N8) viruses. Extrapulmonary dissemination of all HPAI A(H5N8) viruses was primarily detected in the liver (2.3 to 2.5 log TCID_50_), though virus was detected in the brains (2 to 3 log TCID_50_/ml) of Ck/Kumamoto/14-inoculated mice at 3 and 7 dpi (Table 3).

**TABLE 3:** Virus load in different tissues of mice inoculated with HPAI A(H5N8) and A(H5N2) viruses

Subtype	Influenza virus	Mean virus titer (log_10_ EID_50_/ml) in internal organ at indicated dpi:							
		Lung^a^		Brain*^b^,^c^*		Liver*^b^,^c^*		Spleen*^b^,^c^*	
		3	7	3	7	3	7	3	7
H5N2	NP/WA/14	4.04 ± 0.39	1.38 ± 2.75	1 (1)	3 (1)	−^d^	1 (2)	−	−
	SG/MO/15	4.08 ± 0.67	1.75 ± 218	−	−	2.415 (2)	1.33 (3)	−	−
	Tk/MN668/15	1.23 ± 0.78	3.07 ± 0.59	−	−	2 (2)	2.5 (1)	−	−
	Tk/SD/15	4.70 ± 0.082	3.53 ± 0.66	−	−	−	2.5 (1)	−	−
	Tk/ND/15	0.87 ± 0.86	0.7 ± 0.7	−	−	−	−	−	−
H5N8	Md/Korea/14	5.66 ± 0.27	2.25 ± 2.6	−	−	2.5 (2)	2.5 (1)	1 (1)	−
	Ck/Kumamoto/14	2.33 ± 2.69	2.25 ± 2.6	3 (1)	2 (2)	2.5 (1)	2.46 (4)	−	−
	Dk/England/14	4.04 ± 0.82	1.17 ± 2.33	−	−	2.3 (5)	−	−	−
	Gyr/WA/14	1.06 ± 2.12	0.88 ± 1.75	−	−	2.33 (3)	−	−	−
	Ql/CA/14	5.59 ± 0.44	1.75 ± 2.18	−	−	−	−	2.67 (1)	−

a Lung titers shown as mean titer with standard deviation.

b Brain, liver, and spleen titers are averages from positive tissue.

c Parentheses indicate numbers of tissues positive for virus.

d −, virus was not detected in the tissue.

Replication of A(H5N2) viruses in the lungs of C57BL/6 mice varied. Moderate virus titers were observed in the lungs of mice inoculated with NP/WA/14, SG/MO/15, and Tk/SD/15 that waned at 7 dpi (4.04 to 4.70 log TCID_50_/ml at 3 dpi and 1.38 to 3.53 log TCID_50_/ml at 7 dpi). Low lung virus titers (0.87 to 1.23 log TCID_50_/ml) were observed in Tk/MN668/15- and Tk/ND/15-inoculated mice at 3 dpi, though increased virus replication was detected in the lungs of Tk/MN668/15 at 7 dpi (3.07 log TCID_50_/ml). As observed in A(H5N8)-inoculated mice, extrapulmonary dissemination of A(H5N2) viruses was restricted primarily to the liver (1 to 2.415 log TCID_50_/ml), though virus was detected in brain homogenates of 1 NP/WA/14-inoculated mouse at 3 and 7 dpi (1 to 3 log TCID_50_/ml). Compared to HPAI strains, LPAI Ql/CA/14 replicated to higher titers (5.59 and 1.75 log TCID_50_/ml) at 3 and 7 dpi, though virus was not detected in the brains and livers of inoculated mice (Table 3). Collectively, these results indicate that both A(H5N2) and A(H5N8) virus subtypes are capable of replication in mice without prior adaptation, although they cause only mild to moderate disease with little mortality and considerable intraclade variability unrelated to continent of origin.

### Pathogenicity of A(H5N2) and A(H5N8) influenza viruses of HA clade 2.3.4.4 in ferrets.

**FIG 8:**
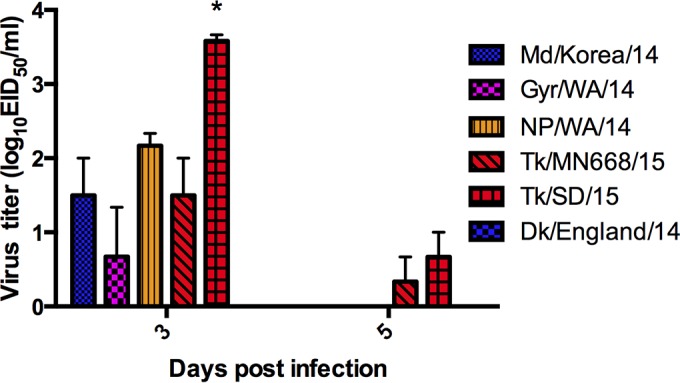
Replication of A(H5N8) and A(H5N2) in the upper respiratory tract of ferrets. Three ferrets were inoculated intranasally with 10^6^ EID_50_ (Md/Korea/14, Dk/England/14, NP/WA/14, Tk/MN688/15, and Tk/SD/15) or 10^7^ EID_50_ (Gyr/WA/14) of virus. Nasal washes were obtained from each ferret at 3, 5, and 7 dpi, and titers were determined in 10-day-old embryonated chicken eggs (EID_50_). Nasal wash titers from ferrets inoculated with Tk/SD/15 (H5N2) were significantly higher (analysis of variance; *, *P* < 0.05) than those from other viruses. Results from donor ferrets are presented; data for 7 dpi are not shown.

Ferrets are an excellent small animal model used to understand the pathogenicity and transmissibility of influenza viruses in mammalian hosts. We evaluated the ability of the clade 2.3.4.4 viruses to replicate in the upper respiratory tract (URT) of ferrets and transmit to direct contacts. Following inoculation, no overt clinical signs, nor an increase in body temperature or loss of body weight, were observed (data not shown). Somewhat unexpectedly, nasal washes collected at 3, 5, and 7 dpi showed minimal virus replication in the URT of donor ferrets (Fig. 8). Viral titers at 3 dpi ranged from undetectable (Dk/England/14) to just 3.75 log TCID_50_ (TK/SD/15). With the exception of trace amounts of virus detected in some ferrets inoculated with the A(H5N2) turkey viruses, all viruses were cleared by 5 dpi. Virus was not detected in the nasal wash samples taken from direct contact ferrets, indicating a lack of transmission via direct contact (data not shown).

Convalescent-phase sera were collected from all ferrets at 21 dpi and tested via hemagglutination inhibition (HI) assay. Donor ferrets showed minimal HI titers (up to 1:20) if seroconversion was detected at all (data not shown), indicating clade 2.3.4.4 viruses to be poorly immunogenic in ferrets, perhaps in part a consequence of limited replication. Convalescent-phase sera from direct-contact animals were negative for HI titers, confirming the results of our nasal wash titration showing that virus was not transmitted between cohoused animals.

## DISCUSSION

Since 1997, H5 HPAI viruses have contributed to the loss of a significant number of domestic poultry in Africa, Asia, and Europe and have continued to cause disease and death in humans in Asia and Africa. In early 2014, a new clade of HPAI H5, clade 2.3.4.4, emerged in eastern China and quickly spread to South Korea and Japan, where large-scale outbreaks of HPAI A(H5N8) resulted in the mass culling of poultry (6, 24). Clade 2.3.4.4 A(H5N8) viruses reached North America in late 2014, being detected in wild birds in Canada and the United States, and quickly reassorted with North American avian influenza viruses, resulting in the appearance of novel HPAI A(H5N2) and A(H5N1) viruses (7–9). Subsequently, HPAI A(H5N2) viruses have spread across the United States, first causing outbreaks in poultry in Western states before spreading east to Midwestern states. Here, we characterize the *in vitro* and *in vivo* phenotypes of a panel of HPAI A(H5N8) and A(H5N2) viruses that represent their dispersal from Asia.

Our phylogenetic analysis shows that the lineage of PB1 and NP gene segments from A(H5N2) strains has been circulating in wild birds from at least 2011. These gene segments were present in a number of subtypes of virus in ducks. Both the PB1 and NP gene segments show high degrees of similarity (>97% and >98%, respectively) to isolates obtained from waterfowl navigating the Mississippi flyway from 2011 to 2014. Interestingly, although the likely direct donors were LPAI viruses in ducks in North America, the PB1 segments phylogenetically related to those of the reassortant A(H5N2) viruses appear to have wide geographic spread. Gene segments from this lineage have been detected across the United States, in Mexico [in HPAI A(H7N3) viruses that caused widespread poultry outbreaks and several human cases in Mexico in 2012 (25, 26)], and in Central America (in viruses from blue-winged teal). Whether this particular lineage of PB1 has unique properties able to support efficient spread and/or replication in poultry remains to be tested.

Results from our HA protein characterization show that A(H5N2) and A(H5N8) viruses of clade 2.3.4.4 are phenotypically similar to avian influenza viruses compared to human influenza viruses. Human influenza A viruses bind predominantly α2,6-SA present on the cell surface, while avian influenza A viruses predominantly bind α2,3-SA (27, 28). Our data show that all clade 2.3.4.4 viruses preferentially bind α2,3-SA, indicating that these viruses have maintained their preference for avian-like receptors throughout their evolution.

The pH of HA activation is an important factor determining the stability and infectivity of influenza A viruses (18, 29, 30). The HA protein of avian influenza A viruses, including HPAI subtype H5, has been reported to be activated at a higher pH than those of mammalian IAV strains (20). Our results show clade 2.3.4.4 viruses to have a relatively high HA activation pH value (approximately pH 6.0), compared to those of human IAV isolates (<pH 5.5). Together with the receptor binding data, these experiments suggest that current clade 2.3.4.4 IAV unable to bind human-like receptors are more susceptible to activation in the mildly acidic environment of the mammalian upper respiratory tract. Thus, in the absence of further adaptation of the HA protein to the mammalian respiratory tract, these viruses appear to pose little risk for sustained transmission in humans. Additionally, we show that all A(H5N8) and A(H5N2) viruses tested as highly susceptible to inhibition by NAIs, indicating that oseltamivir, zanamivir, and peramivir may be an effective treatment in the event of human infection with clade 2.3.4.4 IAV.

Differentiated NHBE cells have been used to assess the ability of IAV strains to replicate in human upper respiratory tract tissues. It has been shown previously that avian influenza viruses replicate inefficiently, compared to human IAV strains, in differentiated NHBE cells (31, 32). Replication of clade 2.3.4.4 A(H5N2) and A(H5N8) viruses was delayed and reached lower titers compared to CA/09(pH1N1) and VN/04 (H5N1). As A(H5N8) and A(H5N2) viruses have receptor binding and acid stability similar to those of VN/04, other factors are likely responsible for their poor growth in these cultures. It is possible to suggest that NA glycoprotein can contribute to the observed effect, as decreased NA activity is important for the adaptation of influenza A(H5N1) virus to human airway epithelium (33). Additionally, studies have shown that the mutation E627K in the PB2 protein, present in VN/04 but missing in clade 2.3.4.4 strains, can enhance the replication of avian influenza viruses in primary human cells (34). Together, these results suggest that HPAI A(H5N8) and A(H5N2) viruses are poorly suited for replication in human respiratory tissues, even less so than some of the other A/goose/Guangdong/1/1996 lineage virus clades.

The ability of HPAI viruses of subtype H5N1 to replicate and cause disease in mice has been well documented (reviewed in reference 35), although there is considerable variation in these traits across all A(H5N1) clades. Our results show HPAI A(H5N8) and A(H5N2) virus inoculations to be relatively benign in mice compared to HPAI H5N1 strains isolated from humans (36, 37). Clade 2.3.4.4 virus strains showed no difference from LPAI Ql/CA/14 (H5N8) virus in their ability to replicate in the lungs of experimentally inoculated mice. We observed little systemic spread of A(H5N8) and A(H5N2) virus strains, though the most extrapulmonary virus dissemination was observed in the liver, with infrequent detection in the brain. Morbidity, mortality, and replication data that we obtained for Md/Korea/14 are similar to those reported in previous studies (38). In other studies assessing archetypal North American clade 2.3.4.4 H5NX virus strains, Gyr/WA/14 (H5N8) and NP/WA/14 (H5N2) were shown to have high mortality at an infectious dose of 10^7^ EID_50_ in BALB/c mice, though differing mortality at lower infectious doses. At 10^6^ EID_50_, NP/WA/14 was 100% lethal for BALB/c mice while Gyr/WA/14 was reported to have a mortality rate of 20% (39). In our hands, NP/WA/14 showed a mildly pathogenic phenotype (20% mortality) in C57BL/6 mice, which could be attributed to the genetic differences between BALB/c and C57BL/6 mouse strains (40, 41). However, the lack of mortality in both BALB/c and C57BL/6 mice strains suggests that A(H5N8) and A(H5N2) viruses have a low-pathogenic phenotype in mammals.

In ferrets, A(H5N8) and A(H5N2) viruses replicated to very low titers (≤3.75 log TCID_50_/ml), were cleared quickly (≤5 dpi), and failed to transmit via the direct contact animals. Previous studies from our laboratory have shown the human isolate VN/04 to be lethal in ferrets with high virus titers in the nasal wash fluid (>5.0 log EID_50_/ml), whereas HPAI viruses isolated from chickens and ducks were variable in disease severity and virus titer in nasal washes (2.6 to 5.8 log EID_50_/ml) collected from ferrets infected under similar conditions (42). While transmission of the A/goose/Guangdong/1/1996 lineage in ferrets has been rarely reported (43), the low replication levels observed with the clade 2.3.4.4 viruses appear to be more clade specific (37, 44). Others have observed the poor replication of these viruses in ferrets (38, 39, 45).

Emerging influenza viruses pose a continual threat to public health, and understanding the ability of novel avian IAV to infect and cause disease in mammals is of utmost importance. The results presented here provide invaluable data characterizing the novel influenza A(H5N2) and A(H5N8) viruses of clade 2.3.4.4 isolated in North America. Our results indicate these viruses to be only moderately pathogenic and poorly adapted for transmission in mammals. The acquisition of genes from North American viruses has maintained the highly pathogenic phenotype of clade 2.3.4.4 A(H5N8) viruses in wild birds and poultry, although it has had no impact on the ability of A(H5N2) viruses to replicate and cause disease in mammals. Continued surveillance is essential for the identification and characterization of further evolution of clade 2.3.4.4 viruses as their geographic distribution expands.

## MATERIALS AND METHODS

### Ethics and biological safety statement.

All animal protocols and experiments were approved and performed under the auspices of the St. Jude Children’s Research Hospital Institutional Animal Care and Usage Committee (SJ IACUC). All *in vitro* and *in vivo* manipulations of HPAI viruses were conducted in a biosafety level 3 enhanced containment laboratory.

### Cell culture.

Madin-Darby canine kidney (MDCK) cells were obtained from the American Type Culture Collection (Manassas, VA) and maintained in 1× minimum essential medium containing 5% fetal bovine serum (FBS) and supplemented with 0.5% penicillin-streptomycin. Vero cells were obtained from the American Type Culture Collection (Manassas, VA) and maintained in Dulbecco’s modified Eagle’s medium supplemented with 5% heat-inactivated fetal bovine serum and 0.5% penicillin-streptomycin. Differentiated normal human bronchial epithelial (NHBE) cells were obtained either from Lonza and cultured according to previously described methods (32) or from MatTek Corporation (Ashland, MA, USA) and cultured according to the manufacturer’s instructions. All cells were maintained at 37°C in 5% CO_2_

### Influenza viruses.

Influenza A/chicken/Kumamoto/1-7/2014 (H5N8), A/mallard/Korea/W452/2014 (H5N8), A/duck/England/30624/2014 (H5N8), A/gyrfalcon/Washington/41088-2/2014 (H5N8), A/Northern pintail/Washington/40624/2015 (H5N2), A/snow goose/Missouri/CC15-84A/2015 (H5N2), A/turkey/Minnesota/11668-1/2015 (H5N2), A/turkey/Minnesota/10777/2015 (H5N2), A/turkey/Minnesota/10915-1/2015 (H5N2), A/turkey/South Dakota/11089-3/2015 (H5N2), A/turkey/North Dakota/11419-1/2015 (H5N2), and LPAI A/quail/California/K1400794/2014 (H5N8) were propagated in the allantoic cavity of 10-day-old embryonated chicken eggs. Allantoic fluid of virus-inoculated eggs was collected at 36 hours postinoculation (hpi), and titers were determined either by 50% egg infective dose (EID_50_) or by 50% tissue culture infective dose (TCID_50_) in MDCK cells as previously described (46).

### HA acid stability.

HA activation pH values were determined by syncytium, hemolysis, and acid inactivation assays. For the syncytium assay, Vero cells were infected with the different H5NX viruses and appropriate control viruses at a multiplicity of infection (MOI) of 5 for 1 h; at 24 h after infection, HA-expressing cells were treated with 1 µg/ml l-tosylamido-2-phenylmethyl chloromethyl ketone (TPCK)-treated trypsin (Worthington Biochemical, NJ, USA) and pH-adjusted phosphate-buffered saline (PBS) buffers for 5 min. Cells were incubated in regular medium containing fetal bovine serum (FBS) for 3 h at 37°C. Cells were fixed and stained with a Protocol Hema 3 kit (Fisher Scientific), and syncytium formation was observed by light microscopy. The pH of activation was determined as the highest pH value at which syncytia were observed. The pH of fusion-induced hemolysis was determined using chicken red blood cells (cRBC). Briefly, virus stock was standardized to 128 hemagglutinin units (HAU) in 50 µl PBS and then diluted with a final 0.4% concentration of cRBC and incubated on ice for 1.5 h to allow the binding of virus to the cells. After centrifugation at 72 × *g* for 3 min, cRBC pellets were resuspended in 100 µl pH-adjusted buffer and incubated at 37°C for 1 h to allow activation of the HA protein and fusion. The absorbance at 405 nm of the hemoglobin-containing supernatants was then measured. To measure the effect of acid exposure on *in vitro* inactivation, prestandardized virus stocks were diluted to 10^6.5^ EID_50_/ml in pH-adjusted PBS solutions and incubated for 1 h at 37°C. The remaining infectious virus titer was then determined by TCID_50_. In hemolysis and acid inactivation assays, the pH values were determined as the point at which a 50% change between maximum and baseline was observed.

### HA cleavability.

HA cleavability was tested using plaque assay and Western blotting approaches. For plaque assay, MDCK cells were infected with a serial 10-fold dilution of viruses for 1 h at 37°C. The medium was then replaced with a mix of low-melting-temperature agar (MP Biomedicals) at a 0.9% final concentration and regular medium with or without 1 µg/ml TPCK-trypsin. Cells were incubated for 2 to 3 days, and plaques were visualized after fixation with 10% formalin followed by staining with 1% crystal violet. To analyze HA cleavage pattern, Vero cells were infected at an MOI of 5, incubated for 24 h at 37°C, and treated or not treated with 5 µg/ml TPCK-treated trypsin for 15 min. Cells were then lysed using radioimmunoprecipitation (RIPA) buffer containing protease inhibitors, and lysates were resolved on 4 to 12% NuPAGE bis-Tris polyacrylamide-SDS gels (Invitrogen) and transferred onto polyvinylidene difluoride (PVDF) membranes. The membranes were blotted using a pool of ferret antiserum to H5 subtypes obtained in the laboratory (Md/Korea/14, Dk/England/14, Gyr/WA/14, NP/WA/14, and VN/04) as well as polyclonal goat anti-H1 antiserum (G618, NR15696; BEI Resources, NIAID, NIH). Bands were visualized using horseradish peroxidase-conjugated anti-ferret (Abcam) and anti-goat (Sigma) antibodies.

### Receptor binding specificity assay.

We used a solid-phase binding assay to measure the receptor binding specificity of the HA protein for α2,3- or α2,6-linked sialic acid. Plates were coated overnight with 10 µg/ml fetuin (Sigma). Virus stocks were standardized to 128 HA units, added to the wells, and incubated overnight at 4°C. After washing, plates were blocked for 1 h at 4°C with PBS containing 0.1% bovine serum albumin (BSA) (Sigma) desialylated by *Vibrio cholerae* neuraminidase treatment. Plates were washed with cold PBS containing 0.01% Tween 80 and incubated with serial dilutions of biotinylated sialylglycopolymers (3′-sialyllactose [3′SL], Neu5Acα2-3Galβ1-4Glc, and 6′-sialyllactosamine [6′SLN], Neu5Acα2-6Galβ1-4Glcβ; Glycotech) for 1.5 h at 4°C. After washing, plates were incubated with horseradish peroxidase-conjugated streptavidin (diluted 1:500; Invitrogen) for 1 h at 4°C. Plates were finally incubated with tetramethylbenzidine substrate (Thermo Scientific), and the absorbance was measured at 450 nm.

### Susceptibility to NAIs.

Susceptibility to NAIs was determined as previously described (47). Briefly, stocks of oseltamivir carboxylate (oseltamivir), zanamivir, and peramivir were prepared in distilled water, filter sterilized, and stored in aliquots at −20°C. Susceptibility to NAIs was assessed in a fluorescence-based assay using 100 µM fluorogenic substrate 2′-(4-methylumbelliferyl)-α-d-*N*-acetylneuraminic acid (MUNANA) (Sigma-Aldrich, St. Louis, MO) (47). Fluorometric determinations were quantified with a Synergy 2 multimode microplate reader (BioTek Instruments, Winooski, VT) based on the release of the fluorescent product 4-methylumbelliferone using excitation and emission wavelengths of 360 and 460 nm, respectively. The concentration of NAI that reduced NA activity by 50% relative to a control mixture with no NAI (IC_50_) was determined using GraphPad Prism 5 software (GraphPad Software, La Jolla, CA).

### Susceptibility to adamantanes.

Molecular markers of adamantane resistance (L26, V27, A30, S31, and G34) were assessed in the transmembrane region of the M2 protein. Nucleotide sequences of the M2 gene were visualized in Geneious v8.1.5 (Auckland, New Zealand) and translated to amino acid sequences where residue identity at positions 26, 27, 30, 31, and 34 was examined.

### Deep amplicon sequencing and phylogenetic analysis.

Allantoic fluid from eggs infected with field isolates was kindly provided by David Stallknecht (University of Georgia). Viral RNA was extracted via the MagMAX 96 total RNA isolation kit (Ambion) on a Kingfisher magnetic particle processor (Thermo Scientific) according to the manufacturer’s instructions. Two-step reverse transcription-PCR (RT-PCR), DNA library preparation, and genomic sequence analysis were done as previously described (46, 48). Briefly, viral RNA was extracted using an RNeasy kit (Qiagen), and cDNA was synthesized via reverse transcriptase PCR using the SuperScript III first-strand synthesis kit (Invitrogen). Influenza A virus gene segments were amplified via multiplex PCR using PCR Supermix HiFi (Invitrogen) with Uni12/13 primers. DNA libraries were prepared using Nextera XT DNA-Seq library prep kits (Illumina) with 96 dual-index bar codes and sequenced on an Illumina MiSeq personal genome sequencer. Full-length IAV gene segments were assembled via a custom workflow protocol developed at St. Jude Children’s Research Hospital (Memphis, TN).

Reference sequences for PB1 (*n* = 66) and NP (*n* = 121) gene segments collected from 2011 to 2015 were downloaded from the Influenza Research Database (http://www.fludb.org). Full cDNA sequences for each segment were aligned with MUSCLE v3.8.3 (49). The phylogenetic relationships of PB1 and NP were reconstructed with RAxML v8.0.0 (50) using a general time-reversible model of nucleotide substitution with a gamma-distributed site variation rate. Bootstrapping was performed (1,000 replicates) to infer the robustness of maximum-likelihood (ML) trees.

### Growth kinetics of influenza virus in differentiated NHBE cells.

Prior to inoculation, basal chamber medium was removed and replaced with 0.5 ml maintenance medium, and the apical chamber was washed 3 times in 1× phosphate-buffered saline solution. NHBE cell inserts were inoculated at a multiplicity of infection of 0.01 in 0.1 ml NHBE cell maintenance medium. Following a 1-h absorption period, the inoculum was removed and the apical chamber of NHBE cell inserts was washed 3 times in 1× phosphate-buffered saline. Time point samples (8, 24, 48, and 72 hpi) were collected by addition of 0.1 ml warmed NHBE cell maintenance medium to the apical chamber for 30 min, after which the medium was collected in cryovials and stored at −80°C until determination of TCID_50_ in MDCK cells.

### Inoculation of mice.

Female 6- to 8-week-old C57BL/6 mice were obtained from The Jackson Laboratory (Bar Harbor, ME). Mice were lightly anesthetized with isoflurane and inoculated intranasally with 10^6^ EID_50_ of virus diluted in sterile PBS. Mice were observed daily for weight loss over a period of 14 days postinfection. At 3 and 7 dpi, 5 mice per group were humanely euthanized and brain, liver, lung, and spleen were collected and stored at −80°C until processing.

### Inoculation of ferrets.

Young adult (6- to 8-month-old) male ferrets (Triple F Farms, Sayre, PA) that were serologically negative by HI assay for currently circulating A(H1N1) and A(H3N2) viruses and influenza B viruses were used in these experiments. Donor ferrets were inoculated intranasally with 10^6^ EID_50_ (Md/Korea/14, Dk/England/14, NP/WA/14, Tk/MN688/15, and Tk/SD/15) or 10^7^ EID_50_ (Gyr/WA/14) of virus in PBS based on stock virus titer. At 1 dpi, naive direct-contact ferrets were introduced into the same cage as an infected donor animal. Ferrets were observed daily for temperature, weight loss, and clinical signs. At 3, 5, and 7 dpi, nasal wash samples were collected from all donor and direct-contact ferrets. At 21 dpi, 5 ml of blood was collected to assess seroconversion of donor and contact animals.

### Statistical analysis.

GraphPad Prism 6 Mac software (GraphPad Software Inc.) was used for statistical analysis. Two-way analysis of variance with Bonferroni’s *post hoc* test was used to compare groups where statistical analysis was applied. *P* < 0.05 was considered to be statistically significant.

### Nucleotide sequence accession numbers.

Sequences have been deposited in GenBank under accession numbers KU895796 to KU895829.
