# Supplementary material for: Novel Highly Pathogenic Avian A(H5N2) and A(H5N8) Influenza Viruses of Clade 2.3.4.4 from North America Have Limited Capacity for Replication and Transmission in Mammals
Source: mSphere. 2016 Apr 6;1(2):e00003-16. doi: 10.1128/mSphere.00003-16 (PMC4894690; doi:10.1128/mSphere.00003-16)
Supplement: Table S1 [file sph002162063st1.docx]

Supplemental Table 1. Nucleic acid sequence identity of clade 2.3.4.4 A(H5N2) virus PB1 gene segments

|  | A/turkey/BC/FAV10/2014\|H5N2 | A/northern pintail/WA/40964/2014\|H5N2 | A/snow goose/MO/CC15-84A/2015\|H5N2 | A/turkey/MN/11668-1/2015\|H5N2 | A/turkey/MN/10777/2015\|H5N2 | A/turkey/MN/10915-1/2015\|H5N2 | A/turkey/ND/11419-1/2015\|H5N2 | A/turkey/SD/11089-3/2015\|H5N2 | A/mallard/MN/UGAI14_2873/2014\|H3N1 | A/mallard/MN/UGAI14_2641/2014\|H4N6 | A/blue winged teal/LA/UGAI14_2115/2014\|H4N8 | A/mallard/MN/UGAI14_3338/2014\|H1N1 | A/mallard/MN/UGAI14_2811/2014\|H3N8 | A/mallard/MN/UGAI14_3313/2014\|H3N2 | A/mallard/MN/UGAI14-2238/2014\|H4N2 | A/mallard/MN/UGAI14-2241/2014\|H4N6 | A/blue winged teal/TX/UGAI14_2059/2014\|H4N6 | A/blue winged teal/TX/UGAI14_3363/2014\|H4N6 | A/quail/CA/K1400794/2014\|H5N8 |
| --- | --- | --- | --- | --- | --- | --- | --- | --- | --- | --- | --- | --- | --- | --- | --- | --- | --- | --- | --- |
| A/turkey/BC/FAV10/2014\|H5N2 |  | 99.82 | 99.82 | 99.69 | 99.74 | 99.69 | 99.60 | 99.76 | 96.60 | 98.15 | 97.76 | 97.89 | 97.93 | 98.37 | 98.02 | 97.98 | 98.02 | 98.11 | 96.39 |
| A/northern pintail/WA/40964/2014\|H5N2 | 99.87 |  | 99.87 | 99.74 | 99.78 | 99.74 | 99.65 | 99.80 | 96.65 | 98.20 | 97.80 | 97.93 | 97.98 | 98.42 | 98.07 | 98.02 | 98.07 | 98.15 | 96.44 |
| A/snow goose/MO/CC15-84A/2015\|H5N2 | 99.82 | 99.87 |  | 99.69 | 99.74 | 99.69 | 99.60 | 99.76 | 96.69 | 98.24 | 97.85 | 97.98 | 98.02 | 98.46 | 98.11 | 98.07 | 98.11 | 98.20 | 96.48 |
| A/turkey/MN/11668-1/2015\|H5N2 | 99.69 | 99.74 | 99.69 |  | 99.78 | 100 | 99.65 | 99.80 | 96.56 | 98.11 | 97.71 | 97.93 | 97.89 | 98.33 | 97.98 | 97.93 | 97.98 | 98.07 | 96.44 |
| A/turkey/MN/10777/2015\|H5N2 | 99.74 | 99.78 | 99.74 | 99.78 |  | 99.78 | 99.69 | 99.85 | 96.51 | 98.07 | 97.67 | 97.89 | 97.85 | 98.28 | 97.93 | 97.89 | 97.93 | 98.02 | 96.39 |
| A/turkey/MN/10915-1/2015\|H5N2 | 99.69 | 99.74 | 99.69 | 100 | 99.78 |  | 99.65 | 99.80 | 96.56 | 98.11 | 97.71 | 97.93 | 97.89 | 98.33 | 97.98 | 97.93 | 97.98 | 98.07 | 96.44 |
| A/turkey/ND/11419-1/2015\|H5N2 | 99.60 | 99.65 | 99.60 | 99.65 | 99.69 | 99.65 |  | 99.74 | 96.38 | 97.93 | 97.54 | 97.76 | 97.71 | 98.15 | 97.80 | 97.76 | 97.80 | 97.89 | 96.35 |
| A/turkey/SD/11089-3/2015\|H5N2 | 99.76 | 99.80 | 99.76 | 99.80 | 99.85 | 99.80 | 99.74 |  | 96.54 | 98.09 | 97.69 | 97.91 | 97.87 | 98.31 | 97.96 | 97.91 | 97.96 | 98.04 | 96.42 |
| A/mallard/MN/UGAI14_2873/2014\|H3N1 | 99.60 | 96.65 | 96.69 | 96.56 | 96.51 | 96.56 | 96.38 | 96.54 |  | 96.91 | 97.88 | 98.36 | 98.41 | 97.17 | 96.91 | 96.87 | 96.87 | 96.95 | 95.02 |
| A/mallard/MN/UGAI14_2641/2014\|H4N6 | 98.15 | 98.20 | 98.24 | 98.11 | 98.07 | 98.11 | 97.93 | 98.09 | 96.91 |  | 98.20 | 98.15 | 98.20 | 98.37 | 98.02 | 97.98 | 98.11 | 98.1 | 96.31 |
| A/blue winged teal/LA/UGAI14_2115/2014\|H4N8 | 97.76 | 97.80 | 97.85 | 97.71 | 97.67 | 97.71 | 97.54 | 97.69 | 97.88 | 98.2 |  | 99.25 | 99.30 | 98.33 | 98.07 | 98.02 | 98.15 | 98.07 | 96.17 |
| A/mallard/MN/UGAI14_3338/2014\|H1N1 | 97.89 | 97.93 | 97.98 | 97.93 | 97.89 | 97.93 | 97.76 | 97.91 | 98.36 | 98.15 | 99.25 |  | 99.78 | 98.46 | 98.20 | 98.15 | 97.98 | 98.20 | 96.31 |
| A/mallard/MN/UGAI14_2811/2014\|H3N8 | 97.93 | 97.98 | 98.02 | 97.89 | 97.85 | 97.89 | 97.71 | 97.87 | 98.41 | 98.20 | 99.30 | 99.78 |  | 98.50 | 98.24 | 98.20 | 98.11 | 98.24 | 96.26 |
| A/mallard/MN/UGAI14_3313/2014\|H3N2 | 98.37 | 98.42 | 98.46 | 98.33 | 98.28 | 98.33 | 98.15 | 98.31 | 97.17 | 98.37 | 98.33 | 98.46 | 98.50 |  | 98.72 | 98.68 | 98.15 | 98.86 | 96.44 |
| A/mallard/MN/UGAI14-2238/2014\|H4N2 | 98.02 | 98.07 | 98.11 | 97.98 | 97.93 | 97.98 | 97.80 | 97.96 | 96.91 | 98.02 | 98.07 | 98.20 | 98.24 | 98.72 |  | 99.96 | 98.77 | 99.03 | 96.09 |
| A/mallard/MN/UGAI14-2241/2014\|H4N6 | 97.98 | 98.02 | 98.07 | 97.93 | 97.89 | 97.93 | 97.76 | 97.91 | 96.87 | 97.98 | 98.02 | 98.15 | 98.20 | 98.68 | 99.96 |  | 98.94 | 98.99 | 96.04 |
| A/blue winged teal/TX/UGAI14_2059/2014\|H4N6 | 98.02 | 98.07 | 98.11 | 97.98 | 97.93 | 97.98 | 97.80 | 97.96 | 96.87 | 98.11 | 97.98 | 98.11 | 98.15 | 98.77 | 98.94 | 98.9 |  | 99.74 | 96.04 |
| A/blue winged teal/TX/UGAI14_3363/2014\|H4N6 | 98.11 | 98.15 | 98.20 | 98.07 | 98.02 | 98.07 | 97.89 | 98.04 | 96.95 | 98.11 | 98.07 | 98.20 | 98.24 | 98.86 | 99.03 | 98.99 | 99.74 |  | 96.13 |
| A/quail/CA/K1400794/2014\|H5N8 | 96.36 | 96.44 | 96.48 | 96.44 | 96.39 | 96.44 | 96.35 | 96.42 | 95.02 | 96.31 | 96.17 | 96.31 | 96.26 | 96.44 | 96.09 | 96.04 | 96.04 | 96.13 |  |
