# Supplementary material for: Novel Highly Pathogenic Avian A(H5N2) and A(H5N8) Influenza Viruses of Clade 2.3.4.4 from North America Have Limited Capacity for Replication and Transmission in Mammals
Source: mSphere. 2016 Apr 6;1(2):e00003-16. doi: 10.1128/mSphere.00003-16 (PMC4894690; doi:10.1128/mSphere.00003-16)
Supplement: Table S2 [file sph002162063st2.docx]

Supplemental Table 2. Nucleic acid sequence homology of clade 2.3.4.4 A(H5N2) virus NP gene segments

|  | A/turkey/BC/FAV10/2014\|H5N2 | A/northern pintail/WA/40964/2014\|H5N2 | A/snow goose/MO/CC15-84A/2015\|H5N2 | A/turkey/MN/11668-1/2015\|H5N2 | A/turkey/MN/10777/2015\|H5N2 | A/turkey/MN/10915-1/2015\|H5N2 | A/turkey/ND/11419-1/2015\|H5N2 | A/turkey/SD/11089-3/2015\|H5N2 | A/blue winged teal/LA/UGAI14_2115/2014\|H4N8 | A/blue winged teal/TX/UGAI14_2059/2014\|H4N6 | A/blue winged teal/TX/UGAI14_3363/2014\|H4N6 | A/mallard/MN/UGAI14-2257/2014\|H4N8 | A/mallard/MN/UGAI14-2238/2014\|H4N2 | A/mallard/MN/UGAI14_3313/2014\|H3N2 | A/mallard/MN/UGAI14_2767/2014\|H4N6 | A/mallard/MN/UGAI14-2241/2014\|H4N6 | A/mallard/MN/UGAI14-2249/2014\|H4N6 | A/quail/CA/K1400794/2014\|H5N8 |
| --- | --- | --- | --- | --- | --- | --- | --- | --- | --- | --- | --- | --- | --- | --- | --- | --- | --- | --- |
| A/turkey/BC/FAV10/2014\|H5N2 |  | 99.93 | 99.03 | 99.87 | 99.87 | 99.93 | 99.87 | 99.87 | 98.53 | 98.73 | 98.46 | 97.80 | 98.06 | 98.60 | 98.53 | 98.60 | 98.66 | 93.59 |
| A/northern pintail/WA/40964/2014\|H5N2 | 99.93 |  | 98.96 | 99.80 | 99.80 | 99.87 | 99.80 | 99.80 | 98.60 | 98.80 | 98.53 | 97.86 | 98.13 | 98.66 | 98.60 | 98.66 | 98.73 | 93.65 |
| A/snow goose/MO/CC15-84A/2015\|H5N2 | 99.03 | 98.96 |  | 98.90 | 98.90 | 98.96 | 98.90 | 98.90 | 97.56 | 97.76 | 97.49 | 96.83 | 97.09 | 97.63 | 97.56 | 97.63 | 97.70 | 92.75 |
| A/turkey/MN/11668-1/2015\|H5N2 | 99.87 | 99.80 | 98.90 |  | 99.87 | 99.93 | 99.87 | 99.87 | 98.40 | 98.60 | 98.33 | 97.66 | 97.93 | 98.46 | 98.40 | 98.46 | 98.53 | 93.45 |
| A/turkey/MN/10777/2015\|H5N2 | 99.87 | 99.80 | 98.90 | 99.87 |  | 99.93 | 99.87 | 99.87 | 98.40 | 98.60 | 98.33 | 97.66 | 97.93 | 98.46 | 98.40 | 98.46 | 98.53 | 93.45 |
| A/turkey/MN/10915-1/2015\|H5N2 | 99.93 | 99.87 | 98.96 | 99.93 | 99.93 |  | 99.93 | 99.93 | 98.46 | 98.66 | 98.40 | 97.73 | 98.00 | 98.53 | 98.46 | 98.53 | 98.60 | 93.52 |
| A/turkey/ND/11419-1/2015\|H5N2 | 99.87 | 99.80 | 98.90 | 99.87 | 99.87 | 99.93 |  | 99.87 | 98.40 | 98.60 | 98.33 | 97.66 | 97.93 | 98.46 | 98.40 | 98.46 | 98.53 | 93.45 |
| A/turkey/SD/11089-3/2015\|H5N2 | 99.87 | 99.80 | 98.90 | 99.87 | 99.87 | 99.93 | 99.87 |  | 98.40 | 98.60 | 98.33 | 97.66 | 97.93 | 98.46 | 98.40 | 98.46 | 98.53 | 93.45 |
| A/blue winged teal/LA/UGAI14_2115/2014\|H4N8 | 98.53 | 98.60 | 97.56 | 98.40 | 98.40 | 98.46 | 98.40 | 98.40 |  | 99.27 | 99.13 | 98.26 | 98.53 | 98.86 | 98.80 | 98.86 | 98.93 | 93.59 |
| A/blue winged teal/TX/UGAI14_2059/2014\|H4N6 | 98.73 | 98.80 | 97.76 | 98.60 | 98.60 | 98.66 | 98.60 | 98.60 | 99.27 |  | 99.60 | 98.20 | 98.60 | 99.06 | 98.86 | 98.93 | 99.00 | 93.79 |
| A/blue winged teal/TX/UGAI14_3363/2014\|H4N6 | 98.46 | 98.53 | 97.49 | 98.33 | 98.33 | 98.40 | 98.33 | 98.33 | 99.13 | 99.60 |  | 97.93 | 98.33 | 98.66 | 98.60 | 98.66 | 98.73 | 93.65 |
| A/mallard/MN/UGAI14-2257/2014\|H4N8 | 97.8 | 97.86 | 96.83 | 97.66 | 97.66 | 97.73 | 97.66 | 97.66 | 98.26 | 98.20 | 97.93 |  | 99.20 | 98.93 | 99.00 | 99.06 | 99.13 | 93.92 |
| A/mallard/MN/UGAI14-2238/2014\|H4N2 | 98.06 | 98.13 | 97.09 | 97.93 | 97.93 | 98.00 | 97.93 | 97.93 | 98.53 | 98.60 | 98.33 | 99.20 |  | 99.20 | 99.27 | 99.33 | 99.40 | 93.72 |
| A/mallard/MN/UGAI14_3313/2014\|H3N2 | 98.60 | 98.66 | 97.63 | 98.46 | 98.46 | 98.53 | 98.46 | 98.46 | 98.86 | 99.06 | 98.66 | 98.93 | 99.20 |  | 99.67 | 99.73 | 99.80 | 93.92 |
| A/mallard/MN/UGAI14_2767/2014\|H4N6 | 98.53 | 98.60 | 97.56 | 98.40 | 98.40 | 98.46 | 98.40 | 98.40 | 98.80 | 98.86 | 98.60 | 99.00 | 99.27 | 99.67 |  | 99.80 | 99.87 | 93.85 |
| A/mallard/MN/UGAI14-2241/2014\|H4N6 | 98.60 | 98.66 | 97.63 | 98.46 | 98.46 | 98.53 | 98.46 | 98.46 | 98.86 | 98.93 | 98.66 | 99.06 | 99.33 | 99.73 | 99.80 |  | 99.93 | 93.92 |
| A/mallard/MN/UGAI14-2249/2014\|H4N6 | 98.66 | 98.73 | 97.70 | 98.53 | 98.53 | 98.60 | 98.53 | 98.53 | 98.93 | 99.00 | 98.73 | 99.13 | 99.40 | 99.80 | 99.87 | 99.93 |  | 93.99 |
| A/quail/CA/K1400794/2014\|H5N8 | 93.59 | 93.65 | 92.75 | 93.45 | 93.45 | 93.52 | 93.45 | 93.45 | 93.59 | 93.79 | 93.65 | 93.92 | 93.72 | 93.92 | 93.85 | 93.92 | 93.99 |  |
